# Paradigms of Lung Microbiota Functions in Health and Disease, Particularly, in Asthma

**DOI:** 10.3389/fphys.2018.01168

**Published:** 2018-08-21

**Authors:** Elliot Mathieu, Unai Escribano-Vazquez, Delphyne Descamps, Claire Cherbuy, Philippe Langella, Sabine Riffault, Aude Remot, Muriel Thomas

**Affiliations:** ^1^Micalis Institute, Institut National de la Recherche Agronomique, AgroParisTech, Université Paris-Saclay, Jouy-en-Josas, France; ^2^Virologie et Immunologie Moléculaires, Institut National de la Recherche Agronomique, Université Paris-Saclay, Jouy-en-Josas, France

**Keywords:** lung, gut, microbiota, physiology, asthma, immunity, gut-lung axis

## Abstract

Improvements in our knowledge of the gut microbiota have broadened our vision of the microbes associated with the intestine. These microbes are essential actors and protectors of digestive and extra-digestive health and, by extension, crucial for human physiology. Similar reconsiderations are currently underway concerning the endogenous microbes of the lungs, with a shift in focus away from their involvement in infections toward a role in physiology. The discovery of the lung microbiota was delayed by the long-held view that the lungs of healthy individuals were sterile and by sampling difficulties. The lung microbiota has a low density, and the maintenance of small numbers of bacteria seems to be a critical determinant of good health. This review aims to highlight how knowledge about the lung microbiota can change our conception of lung physiology and respiratory health. We provide support for this point of view with knowledge acquired about the gut microbiota and intestinal physiology. We describe the main characteristics of the lung microbiota and its functional impact on lung physiology, particularly in healthy individuals, after birth, but also in asthma. We describe some of the physiological features of the respiratory tract potentially favoring the installation of a dysbiotic microbiota. The gut microbiota feeds and matures the intestinal epithelium and is involved in immunity, when the principal role of the lung microbiota seems to be the orientation and balance of aspects of immune and epithelial responsiveness. This implies that the local and remote effects of bacterial communities are likely to be determinant in many respiratory diseases caused by viruses, allergens or genetic deficiency. Finally, we discuss the reciprocal connections between the gut and lungs that render these two compartments inseparable.

## Paucity and Continual Renewal: Two Main Characteristics of the Lung Microbiota

The lung microbiota has a low density, at 10^3^–10^5^ CFU/g of lung tissue, as estimated by culture methods, in mice (Remot et al., 2017). Human lungs harbor approximately 2.2 × 10^3^ bacterial genomes per cm^2^ (Hilty et al., 2010). The maintenance of a small bacterial community in the lungs seems to be a hallmark of good health. The microbial population of the lung is smaller than that of the colon, which is one of the most densely populated ecosystems in the body, with a microbiota of up to 10^11^ CFU/g of luminal content. However, the microbial population of the lung is equivalent to that of the duodenum (around 10^4^ micro-organisms per mL of content) (Aron-Wisnewsky et al., 2012).

**FIGURE 1:**
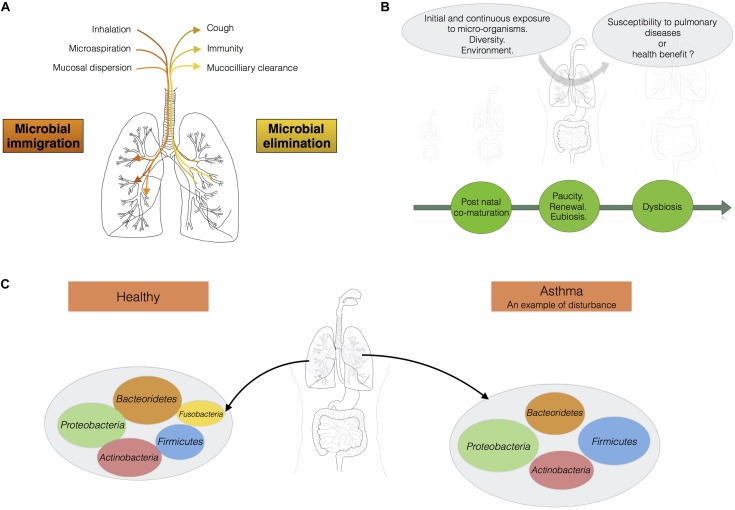
The lungs should be considered as an ecosystem with its own microbiota. **(A)** Maintenance of balance in the lung microbiota: the communities of micro-organisms in the lungs are shaped by microbial immigration and elimination [adapted from (Dickson et al., 2014)]. **(B)** From birth onwards, the lungs are continually exposed to diverse micro-organisms. This diversity of bacterial exposure, the environment and any treatments administered may play a fundamental role in determining susceptibility to pulmonary disease. **(C)** In a healthy individual, the load of micro-organisms in the lungs is equivalent to 10^3^–10^5^ bacteria per gram. Proteobacteria, Firmicutes, and Bacteroidetes are the main phyla present. Asthma is associated with a shift in the lung microbiota toward greater diversity and species richness.

The micro-organisms comprising the microbiotas of both the gut and lungs enter the body via the oral cavity. Bacteria travel to the lungs suspended in air and on microparticles in secretions, such as saliva, whereas the bacteria colonizing the intestine may also be present in ingested food. The lung microbiota disperses from the oral cavity, and a constant balance is maintained between microbial immigration and elimination (Morris et al., 2013; Dickson and Huffnagle, 2015; Venkataraman et al., 2015; **Figure 1A**). The immigration of micro-organisms results from mucosal dispersion, micro-aspiration, and inhalation (Dickson and Huffnagle, 2015; Segal et al., 2016). Humans breathe through both the nose and mouth, whereas mice are obligate nasal breathers (Singh et al., 2017). Anatomical features and natural modes of breathing influence the arrival of microbes in the lung. The elimination of micro-organisms is governed by mucociliary movements, coughing, and host immunity. During lung disease, the balance between immigration and elimination is disturbed, resulting in alterations to the lung microbiota, with bacteria displaying competitive advantages becoming predominant (Dickson and Huffnagle, 2015; **Figure 1B**). Venkataraman et al. (2015) has suggested that the degree of departure from neutrality is correlated with disease severity in the lung. “Neutrality” refers to the neutral biodiversity theory, according to which, all micro-organisms have similar opportunities of reaching and growing in a specific environment, but also of being lost from that environment. In this model, the microbial communities are not selected by the resources accessible in the environment or the inter-species interactions leading to the creation of multiple niches in an environment (Venkataraman et al., 2015). In healthy conditions, the lung microbiota disperses neutrally from the mouth, whereas lung diseases are associated with stronger selection for specific microbes. The lung microbiota seems to be shaped by continual waves of intrusion and expulsion in healthy humans, because the installation of dominant bacterial communities tends to be restricted to pathological contexts. The overgrowth of bacterial species, leading to a decrease in the species richness of the lung microbiota, is associated with the progression of diseases such as cystic fibrosis and with infections (de Dios Caballero et al., 2017; Zemanick et al., 2017).

The low density and continual renewal of the lung microbiota are not inconsistent with a major impact on respiratory health and homeostasis. The duodenal microbiota plays a key role in iron uptake and storage in the context of digestive physiology (Deschemin et al., 2016). The transit of bacteria through a tissue may have a long-term impact on immunity, as shown for the gut (Hapfelmeier et al., 2010; Tomas et al., 2013). A low density and continual renewal are, thus, intrinsic properties of the microbial stimulation of the lungs that may modify lung immunity and physiology.

## Composition of the Lung Microbiota

The microbial community is continually being renewed and replaced, but most of the microbes involved in these fluxes belong to four phyla: Bacteroidetes, Firmicutes, Proteobacteria, and Actinobacteria (Dickson and Huffnagle, 2015; Segal et al., 2016; Yu et al., 2016; **Figure 1C**). In healthy individuals, *Prevotella*, *Streptococcus*, *Veillonella*, *Neisseria*, *Haemophilus*, and *Fusobacterium* are the most abundant genera in the lungs (Hilty et al., 2010). The four main phyla present are identical in humans and mice. However, Bacteroidetes and Firmicutes predominate in humans, whereas Proteobacteria and Firmicutes predominate in mice. The respiratory microbiota has also been described in domestic animals (cats, dogs), and in farm animals (pigs, sheeps, and calves), which can serve as relevant translational models for humans (Ericsson et al., 2016; Glendinning et al., 2016; Nicola et al., 2017; Siqueira et al., 2017; Vientos-Plotts et al., 2017a).

The lung microbiota displays greater spatial variation between than within individuals, and differences between sites in the lung (position relative to the alveoli) result from waves of elimination/immigration and differences in distance from the mouth, which serves as the source of the community (Dickson and Huffnagle, 2015). The analysis of low-density communities is a methodological challenge. In low-density samples, contaminant (or non-related) DNA can predominate over the true sample DNA, creating a shift in the microbial profile obtained. A major impact of extraction methods on relative abundance and bacterial representation has been reported at densities below 10^6^ bacteria per mL of sample (Biesbroek et al., 2012). The analysis of low-density communities can be challenging, and bias is likely, so particular attention must be paid to the choice of the method and data interpretation, particularly for the lung microbiota.

Due to the high degree of variability between individuals, there is currently no consensus concerning the definition of a “typical” microbiota, constituting a state of homeostasis between the microbiota and the host cells. Moreover, it remains unclear whether specific bacteria or microbiota profiles could serve as markers or drivers of good lung health. There are probably beneficial lung bacteria, as already suggested in the intestine for commensal organisms such as *Faecalibacterium prausnitzii* (Miquel et al., 2015).

During lung diseases, such as asthma in particular, a shift in the lung microbiota is observed that may be seen as an imbalance or dysbiosis (Hooks and O’Malley, 2017). This shift in the lung microbiota may also be interpreted as the emergence of particular dominant bacteria in lungs. It remains a matter of debate whether we should be talking about dysbiosis, stable colonization, or infections of the lungs. The function and causal role of this dysbiosis in the onset and outcome of asthma remain unclear. An analysis of BAL from children with severe asthma has shown a phylum distribution different from that in control subjects, with, in order of abundance, Proteobacteria, Firmicutes (mainly *Streptococcus*), Bacteroidetes (mainly *Prevotella*), and Actinobacteria (Hilty et al., 2010). At genus level, *Staphylococcus* and *Haemophilus* are more abundant in asthma sufferers, whereas *Prevotella* is more abundant in controls (Hilty et al., 2010). The lung microbiota is more diverse and abundant in some subjects with asthma (Hilty et al., 2010; Huang et al., 2011; Goleva et al., 2013; Fujimura and Lynch, 2015). As for all microbial communities, measurements of abundance and diversity will improve our understanding of the ecological mechanisms underlying health and the management of endogenous communities (Shade, 2017). We now need to determine the mechanisms underlying the maintenance of lung microbial communities, to find ways of preventing respiratory diseases, such as asthma.

## The Physiological Characteristics of the Lungs Influencing the Homeostasis Between the Lung and Its Microbiota

**FIGURE 2:**
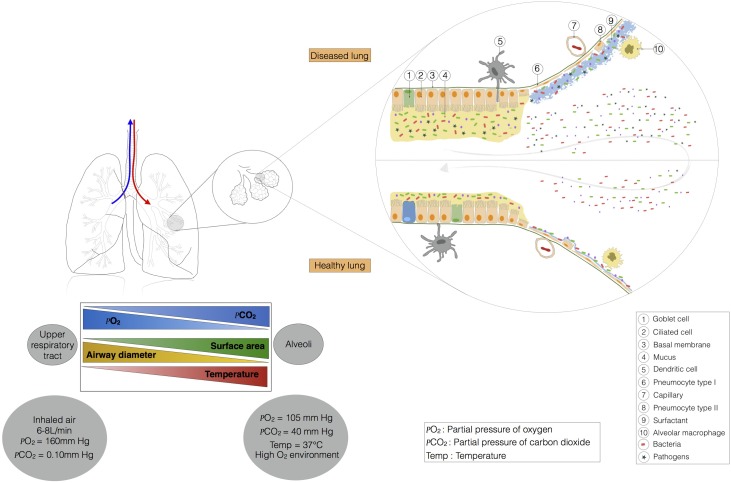
This ecosystem is shaped by lung physiology, which changes radically from the upper respiratory tract to the alveoli. Indeed, in the trachea, the airway has a diameter of about 15 mm, and the partial pressures of oxygen and carbon dioxide are similar to those in the external environment. Temperature and contact area are low. Moving toward the alveoli, temperature, contact area, and the partial pressure of carbon dioxide increase, whereas the partial pressure of oxygen and airway diameter decrease. The barometric pressure in the lung is dependent on pulmonary ventilation. At steady state (no air flow) the pressure is similar in the alveoli and in the external environment. During inspiration and expiration, the pressure falls, and increases, respectively. The airway environment also changes and may favor the selection of certain bacteria, leading to the installation of pathogens.

Microbial immigration and elimination govern the composition of a healthy lung microbiota, but, conversely, certain physiological features of the respiratory tract may favor the installation of a dysbiotic microbiota, influencing susceptibility to pulmonary diseases. The main function of the lungs is to transfer oxygen from the air into the bloodstream, in exchange for CO_2_. The action of the diaphragm increases lung volume, decreasing pressure in the lung and causing air to enter (**Figure 2**). Temperature varies along the respiratory tract, from the mouth and nose to the alveoli. The respiratory system gradually warms the air to 37°C. The gradients of pressure and temperature between the upper respiratory tract and the alveoli may affect bacterial communities. The pulmonary epithelium is composed of ciliated and secretory cells, but is not continuous from the upper respiratory tract to the alveoli (Evans et al., 2010; **Figure 2**). Indeed, in the large bronchi, the mucous and serous cells are located in a submucosal gland that produces mucus (not shown in **Figure 2**). Moving toward the bronchiole, mucus is produced by club and goblet cells. Type I and II pneumocytes form the alveolar epithelium, which secretes a surfactant rather than mucus. Mucus is a gel consisting mostly of water and complex polysaccharides, such as mucins (Evans et al., 2010). MUC5AC (from goblet cells) and MUC5B (from submucosal glands) are the dominant mucins in human airways, together with MUC2, which is produced in only small amounts (Proud and Leigh, 2011). Water and mucins form a thin mobile layer that is supported by a pericilliary layer covering the cilia. In a healthy individual, the mucus layer provides an effective defense against epithelial injury, but excessive mucus production contributes to obstruction in several respiratory diseases (e.g., pneumonia, asthma, chronic obstructive pulmonary diseases, cystic fibrosis).

This obstruction may lead to the production of even more mucus, making it increasingly difficult for the cilia to transport the mucus out of the lungs. A longer residence time of mucus in the airways may favor the selection of certain bacteria with a high tropism for mucus, leading to the installation of pathogens (Flynn et al., 2016). Flynn et al. (2016) have shown that some bacteria present in sputum make use of mucins to produce metabolites, such as propionate in particular, which can be used by *Pseudomonas aeruginosa*. The maintenance or selection of the microbiota is also determined by the nutrient sources available in a particular ecological niche. In the gastrointestinal tract, nutrient sources capable of supporting microbial growth are present at high abundance (due to the breakdown of food). The microbes of the intestinal tract are, therefore, commensal, because they can share the food we eat. By contrast, most of the nutrients available in the lungs are derived from host compounds, such as Igs, cytokines, defensins, lactoferrins, and mucins (Flynn et al., 2016). Lung epithelial cells express various innate sensors on their membranes and in their cytoplasm (TLR, NLR, CLR, and PAR) (Jung et al., 2016) that can detect microbes and activate molecular cascades in host cells, triggering the induction of tolerance or inflammation (Hammad et al., 2009; Di Stefano et al., 2017). For example, many studies of the role of TLR4 in asthma have been performed with the TLR4 agonist LPS. Bottomly’s group has shown that sensitisation to inhaled inert proteins requires LPS and the TLR4 signaling pathway (Eisenbarth et al., 2002). The dose of LPS is critical, as low doses break tolerance and exacerbate the signs of asthma (Eisenbarth et al., 2002), whereas high doses prime protective responses (Hammad and Lambrecht, 2008; Rodriguez et al., 2014). These differences in lung biotic (cell layers) and abiotic (temperature, pressure, mucus, surfactant) environments may have a major impact on the installation and location of bacterial communities, particularly if they lead to certain bacteria being selected and becoming predominant in disease processes.

## Progressive and Sequential Installation of the Microbiota in the Lungs After Birth

### Effect of Delivery Mode on the Lung Microbiota

The airway microbiota sampled by tracheal aspiration is similar in preterm infants born by Cesarean section and in those born by the vaginal route, and consists predominantly of Proteobacteria and Firmicutes during the first few days of life (Lal et al., 2016). However, despite the limited impact of the mode of delivery on the composition of the nasopharyngeal microbiota immediately after birth, subtle differences in respiratory microbial development may appear over time between children born by the vaginal route and those delivered by Cesarean section (Bosch et al., 2016). It therefore remains unclear whether mode of delivery has a strong or weak influence on the composition of the lung microbiota in babies (Blaser and Dominguez-Bello, 2016; Chu et al., 2017), but the microbiota of the mouth and, by extension, the lungs, may be subtly influenced by delivery conditions.

### Post-natal Co-maturation of the Microbiota and Lungs

The lungs of newborn humans face daily challenges in the form of diverse new microbes and environmental components, including allergens (**Figure 1B**), and the postnatal period has a major impact on future health. The development of the lungs, like that of other organs, is not complete at birth. The lungs begin to develop, with the formation of the branching structure, during the embryonic and fetal periods. Lung development is then completed during the postnatal period, when the terminal units of the branching structure, the alveoli, finish developing, along with the vascular system. The immunological development of the lungs also follows a chronological pattern, beginning in the embryo and continuing through the post-natal period, with the sequential arrival of monocytes/macrophages and granulocytes/neutrophils, the recruitment of type 2 innate cells and the accumulation of DC, B, and T cells until weaning (Drajac et al., 2017). Biesbroek et al. (2014) analyzed the development of the microbiota in the upper respiratory tract (nasopharyngeal samples) at different time points during the first 2 years of life (1.5, 6, 12, and 24 months) in healthy individuals. They revealed different microbiota profiles between the members of the cohort at ages as young as 1.5 months. Early colonization with specific micro-organisms influences the subsequent stability of the upper respiratory tract microbiota and susceptibility to pulmonary infection (Biesbroek et al., 2014).

Singh et al. (2017) analyzed the sequential arrival of bacteria in the lungs of mice aged between one and 8 weeks. They observed dynamic changes in mouse lungs over this period. For example, the genus *Streptococcus* predominated when the mice were 2 weeks old, whereas *Lactobacillus* and *Achromobacter* were the most abundant genera when the mice were 4 weeks old (Singh et al., 2017). The phyla Firmicutes and Gammaproteobacteria arrive in the lungs before Bacteroidetes (Gollwitzer et al., 2014). Viable bacteria begin to arrive in the lungs of mice after birth (Remot et al., 2017), and the number of pulmonary bacteria significantly increases until weaning and adulthood (Gollwitzer et al., 2014; Remot et al., 2017). The diversity and abundance of cultivable bacteria also increases with age, from birth to adulthood, in mice (Remot et al., 2017; Singh et al., 2017). Most descriptions of the progressive installation of the lung microbiota after birth relate to mice, but this pattern is consistent with the overall maturation of human microbiotas, which is particularly well described for the gut, with a progressive acquisition of diversity and stability over the first 3 years (Lozupone et al., 2012).

### Hygiene Theory and Asthma

According to the hygiene theory, lower levels of exposure to microbes in urban than in rural areas result in a higher incidence of allergy and asthma (Olszak et al., 2012; Marsland, 2013; Segal et al., 2014). Exposure to LPS, a component of Gram-negative bacteria, decreases asthma levels in mice by suppressing the activation of epithelial and DCs via induction of the ubiquitin-modifying enzyme A20 (Schuijs et al., 2015). Several cross-sectional studies in different countries have compared the prevalence of asthma, hay fever and allergic sensitization in children living in farming and non-farming environments (Kilpelainen et al., 2000; Riedler et al., 2000; Portengen et al., 2002; Douwes et al., 2007). They showed that children born and raised in a farming environment were less prone to the development of atopic symptoms and asthma later in life. This protective effect was even stronger in adults that had remained in the farming environment (Douwes et al., 2007). These observations support the hypothesis that exposure to a wide range of diverse microbial signals during the first few months of life has a major impact on susceptibility to the development of asthma.

## Impact of the Lung Microbiota on the Adaptive and Innate Immune Capacities of the Lungs

**FIGURE 3:**
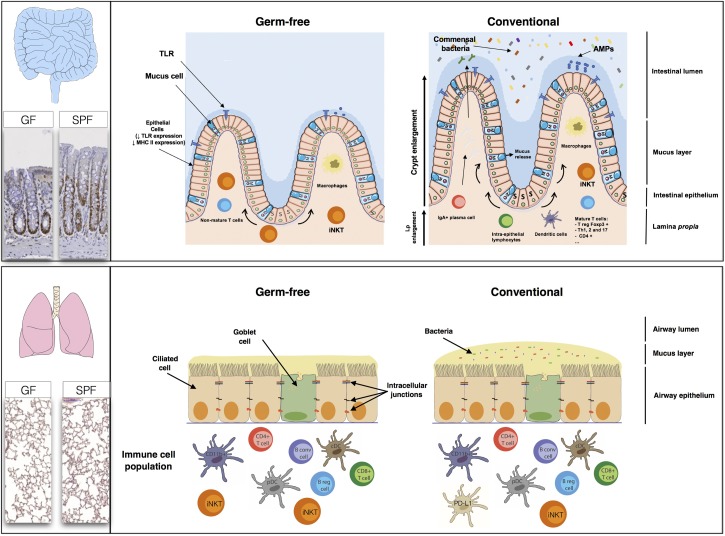
The high rate of renewal of the intestinal epithelium and the diversity of the populations of cells in the intestinal mucosa, comprising immune, absorptive and secretory cells, create a large, flexible arsenal of innate, and acquired defenses against a dense microbiota. Studies in germ-free (GF) mice have shown that normal gastrointestinal tract development is dependent on the presence of a commensal microbiota. The epithelial cell monolayer is linked and maintained by the apical junctions, consisting of adherens, and tight junctions (Miyoshi and Takai, 2005). The regulation of apical junctions is crucial, to prevent the translocation of bacteria, or molecules through the cell monolayer. The lungs appear to be less affected by the absence of a microbiota than the gut. The levels of B cells, T cells, conventional dendritic cells (cDC), and plasmacytoid dendritic (pDC) cells are similar in GF mice and SPF mice. The only major differences between GF and SPF mice are that PD-L1 expression is stronger in SPF mice, whereas GF mice have higher iNKT levels (both in the lungs and the gut) than SPF mice.

The lung may display a similar homeostasis to the gut, in terms of the co-evolution of eukaryotic and prokaryotic cells, and dialog between these cells. In the gut, the microbiota is involved in digestion, energy provision, maturation of the immune system and shaping the structure and modulating the absorption and secretion functions of the epithelium. However, much less is known about the physiological effects of the lung microbiota (**Figure 3**).

### Immune System

Many studies on the gut have reported changes to the immune phenotype, with deficits of both the innate and adaptive immune components of the intestinal mucosa in GF mice. Several bacterial species have been shown to have different modulatory effects on the host immune system, highlighting the need for specific bacteria within a given developmental window for the normal patterning of host immunity (Olszak et al., 2012; Ubags and Marsland, 2017). GF mice have a thinner lamina propria, with fewer resident mature immune cells, and the presence of a complex microbiota triggers the proliferation, differentiation, and maturation of immune cells, leading to an increase in microbiota-selected IgA^+^ (immunoglobulin A) plasma cells or T-cell subsets, such as T_reg_ Foxp3, Th1 (T-helper), Th2, and Th17 lymphocytes (Macpherson and Uhr, 2004; Gaboriau-Routhiau et al., 2009; Ivanov et al., 2009). Some of these mechanisms are mediated by receptors, such as TLRs, which are essential for homeostasis in the intestinal epithelium (Rakoff-Nahoum et al., 2004).

Studies comparing GF and SPF mice during the first few weeks of life have shown that microbial colonization of the lung has no major effect on the subsets of immune cells present. The levels of B and T (CD4 and CD8) cells, conventional CD11b^+^ and CD103^+^ DCs and pDCs are similar in the presence and absence of a microbiota (Gollwitzer et al., 2014; Remot et al., 2017). However, the lungs of GF mice contain 2.5 times as many iNKT than those of SPF mice. Interestingly, when the lungs of GF neonates were exposed to a conventional microbiota, iNKT cell levels were found to be similar to those in SPF mice (Olszak et al., 2012). The bacterial communities in the lung modulate the expression of certain innate immunity genes, resulting in higher levels of IL-5 (interleukin), IL-10, IFNγ, and CCL11 in SPF mice. The level of expression of PD-L1 on CD11b^+^ DCs and the frequency of FoxP3^+^CD25^+^ T_reg_ cells are also higher in the lungs of SPF neonates (Gollwitzer et al., 2014). Comparisons of SPF, GF, conventional, and antibiotic-treated rodents can provide information about the modification of pattern recognition receptor expression (PRRs: TLR, NLR, CLR, and PAR) in the lungs by the microbiota. Segal et al. (2016) recently showed that the TLR4 responses of AMs were influenced by the composition of the lung microbiome.

### Mucus

Germ-free mice have a thinner mucus layer in the gut than conventional mice with a complex microbial ecosystem impregnating the mucus layer close to the epithelial cells and AMPs. These small peptides keep bacteria off of the epithelium and limit bacterial growth. AMP production may be modulated in a specific manner by the microbiota, as reported for beta-defensins, or may be microbiota-independent, as described for lysozymes (Pütsep et al., 2002; Gallo and Hooper, 2012). Mucus production is a dynamic process that may be accelerated or slowed by the microbiota, as shown in the intestine (Wrzosek et al., 2013). Few data are available for the lung, but *muc5ac* (the main mucin in the lung) mRNA levels have been shown to be higher in SPF mice than in their GF counterparts (Remot et al., 2017). Yun et al. (2014) have also reported lower levels of mucus production by the lungs when bacteria are absent or present at low abundance. The production of mucus by the lungs therefore appears to be shaped by the lung microbiota, through as yet unknown mechanisms. By modulating mucus production, the microbiota may modify the barrier function of the respiratory epithelium or favor invasion by mucus-degrading bacteria in some diseases.

### Tissue Organization

As illustrated in **Figure 3**, microbes can greatly modify the morphology of the intestinal epithelium (Cherbuy et al., 2010; Jones et al., 2013; Tomas et al., 2013), with some commensal strains of *Escherichia coli* having morphogenic activity (Tomas et al., 2015). The morphogenic effects of the microbiota are more subtle in the lungs. No marked differences in pulmonary structure, epithelium thickness, or bronchus number are observed between lungs with and without a microbiota, but the number of alveolae is greater in the presence of a microbiota (Yun et al., 2014; Remot et al., 2017).

### Tolerance

The differences (in the immune system, mucus, and epithelium) between GF and SPF animals are less marked in the lungs than in the gut, but microbes in the lung clearly modulate susceptibility to respiratory disorders. During their development, the lungs of neonates are exposed to bacterial stimuli that may affect the maturation of the pulmonary tissue, conferring susceptibility to lung disorders, such as allergic asthma (Sabatel et al., 2017; Ubags and Marsland, 2017). Lung microbes have been shown to promote tolerance, potentially accounting for the hypersensitivity of neonates to allergens. Indeed, during the first 2 weeks of life, mice display high levels of allergic airway inflammation, producing large amounts of IL-4, IL-5, and IL-13 following treatment with HDM allergen. The overproduction of these Th2 cytokines in the lungs of neonates after HDM treatment is coupled to an increase in the proportion of FoxP3^+^CD25^+^ T_reg_ and CD11b^+^ DCs and an increase in expression of the surface ligands PD-L1, PD-L2, and CD40. This allergic airway inflammation is significantly attenuated in adult mice. Gollwitzer et al. (2014) suggested that the tolerance of mice to HDM allergen might improve through the expression of PD-1 on lung T_reg_ cells, together with its ligand PD-L1 on CD11b^+^ DCs. The postnatal susceptibility period strongly affects responsiveness to aeroallergens. It is also correlated with the installation of the stable microbiota in the lungs and the final stages of pulmonary development. Our work has helped to demonstrate that the administration of primary lung-colonizing strains before HDM treatment affects responsiveness to aeroallergens in mice (Remot et al., 2017). Microbial stimulation of the lung at a given time may, therefore, promote allergen tolerance. The impact of the lung microbiota on lung immune capacity in this window of opportunity thus appears to play a key role in susceptibility to the development of allergic diseases, such as asthma, in adulthood.

## The Gut-Lung Axis

**FIGURE 4:**
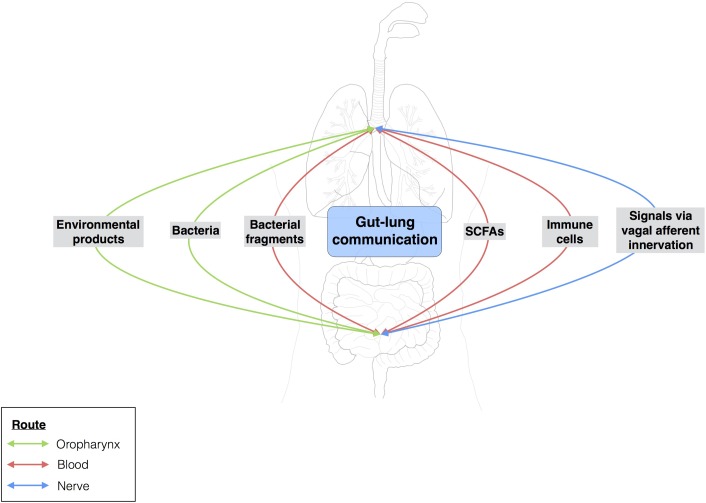
Gut-lung communication. Extensive studies have assessed the local impact of a particular microbiota on organs. Over the last few years, researchers have become interested in the possible crosstalk between different sites within the body. The gut-brain axis is the best known example of this, but the gut-lung axis has also attracted attention. Few data are available for the gut-lung axis, but environmental products and bacteria can be translocated from the gut to the lung (and vice versa) via oropharynx reflux and micro-aspiration. The bloodstream may also serve as a route of communication between the lungs and the gut. Changes to the gut microbiota, such as the modulation of segmented filamentous bacterial load, may influence the outcome of *Staphylococcus aureus* pneumonia in the lungs.

The gut-lung axis comprises the anatomical, systemic, and nervous system connections mediating reciprocal exchanges of microbial signals between the lungs and the gut (**Figure 4**). One of the connections between the gut and the lung involves the translocation of bacteria via oropharynx reflux. Indeed, the human body experiences multiple reflux events (especially in pathological conditions) that can transport different bacterial communities from the digestive tract to the upper respiratory tract, with the bacteria then translocated to the lungs by micro-aspiration. However, this does not mean that bacteria from the digestive tract can reside in the lungs. Moreover, bacteria and bacterial fragments may also be translocated in the blood and lymph. The blood and lymph play a major role in the migration of immune cells to distal sites. For example, bacteria from the gut taken up into DCs and macrophages through phagocytosis prime naïve B and T cells, which may then migrate to the lungs or return to the gut (Bingula et al., 2017).

In physiological conditions, the gut microbiota of old mice (18–22 months of age) may influence inflammation in the lungs and the immune senescence of macrophages (Thevaranjan et al., 2017). High levels of circulating bacterial toxins after fecal transplantation have also been shown to result in low levels of tight junction gene expression and lethal pulmonary damage (Ji et al., 2014). The bacterial taxa implicated are mostly clostridial species. The existence of these connections was suggested by numerous epidemiological observations, but the underlying mechanisms linking gut and lung physiology remain elusive and hypothetical.

Short-chain fatty acids, which are produced in large amounts by some commensal bacteria, can act as substrates for host cells and as signaling molecules between tissues. The metabolic profiling of the microbial community of the lungs is incomplete and the role of SCFAs as organizers of endogenous lung microbial communities, local actors in the respiratory epithelium and immunity, and systemic mediators remains unclear. The importance of these bacterial metabolites and bacteria in the gut for host local immunity has been studied in detail. However, little is known about their impact on distal immunomodulation, in the lungs. For example, few data are currently available concerning immunomodulation by the microbiota at distal sites. However, one study has shown that mice lacking SFB in the gut are prone to more severe *Staphylococcus aureus* pneumonia. The bacterial load in the lungs of mice lacking SFB is 21 times higher than that of mice with SFB in the gut. This higher bacterial load is accompanied by a modulation of pulmonary Th17 immunity, with lower levels of IL-22 in BAL fluid (Gauguet et al., 2015). Modulation of the composition of the gut microbiota in mice regulates the immune response of the respiratory tract and alters susceptibility to pulmonary influenza infections (Ichinohe et al., 2011; Rosshart et al., 2017).

A recent review described a pathogenic link between the microbiota and the gut-lung axis (Budden et al., 2017). We will therefore focus here on the gut-lung axis in asthma. It has been shown that the progressive and sequential acquisition of the gut microbiota after birth determines subsequent susceptibility to allergy (Arrieta et al., 2015; Fujimura et al., 2016; Stokholm et al., 2018). During the perinatal period, the gut microbiota plays a key role in determining future tolerance, the maturation of immunity and asthma risk. It is not yet possible to estimate the relative contributions of the establishment of the lung and gut microbiotas in humans. The gut-lung axis may also be affected by the dual location of some environmental allergens, weakening the barriers in both the gut and the lung and stimulating a cascade of detrimental inflammatory pathways. In particular, HDM is both inhaled and ingested, and its Der p1-associated allergen is detected in the gut, where it can impair the barrier function of the intestine (Tulic et al., 2016). The manipulation of both gut and lung microbiotas, by oral supplementation or nasal administration, may be beneficial, favoring homeostatic feedback and decreasing risk (Arrieta et al., 2015; Remot et al., 2017; Durack et al., 2018). Many studies have tested the hypothesis that pre- and probiotics can protect against asthma, but the possible mode of action has yet to be elucidated in mice. The use of a large-animal model to study the effects of oral probiotics on respiratory microbiota may be of interest as a potential translational model for humans (Vientos-Plotts et al., 2017b). A few preclinical data for humans supporting the use of specific products are available (Mennini et al., 2017). Conversely, specific diets may also increase the risk. In mice a HFD, and the consumption of saturated fatty acids (palmitic acid) in particular, has been shown to increase the proportion of circulating monocytes and AMs in the lung. Moreover, the combination of a HFD with HDM stimulation results in airway responsiveness, inflammatory cell levels, goblet cell hyperplasia, total cell number, levels of neutrophils, eosinophils, and macrophages and of IL-13, IL-17A, and 1L-1β production greater than those in mice fed a HFD in the absence of HDM stimulation (Tashiro et al., 2017). Marsland’s group demonstrated that the metabolism, by the gut microbiota, of dietary fiber influences allergic airway disease and haematopoiesis (Trompette et al., 2014). Mice fed a high-fiber diet had high levels of SCFA in the blood and were protected against HDM-induced asthma, whereas a low-fiber diet resulted in lower SCFA levels and a higher frequency of asthma. Protection was associated with the beneficial effects of propionate, which modified haematopoiesis in a GRP41-dependent manner, particularly in terms of DC levels and Th2 differentiation. Similar correlations between a low incidence of asthma and changes in the intestinal microbiota after fiber intake have been reported in humans (De Filippo et al., 2010; Marsland et al., 2015).

There is currently no consensus definition of a “healthy” lung microbiota as a function of age, diet, or environment and, given the considerable interindividual variability of the gut microbiota, we are still far from a definition of the best gut/lung microbiota configuration to optimize digestive and respiratory health. It should also be noted that the influence of the gut microbiota on distal immunity is not restricted to the lung. The gut microbiota has been shown to affect hepatic immune responses (Chou et al., 2015), and to shape the immune pancreatic environment (Ryu and Stappenbeck, 2015). All these studies support the hypothesis that the host microbiota can affect the “common mucosal immune system.”

## Conclusion

Interest in the lung microbiota has steadily increased over the last decade, and it is now widely accepted that the lungs harbor bacterial communities. Like those of the gut, the micro-organisms of the respiratory microbiota play a role in health and diseases, such as asthma. We are gradually learning more about the densities of the various members of the community, their sources and their dispersion throughout the branched structure of bronchi. More intensive studies of the local impact of the lung microbiota and the pathways involved in gut-lung communication are required. A number of questions remain to be addressed. Are bacteria or their products translocated via the oropharynx reflux or via the blood in pathogenic and physiological conditions? How intense are immune cell exchanges between the two sites, and what impact do they have? What do lung bacteria use as an energy source? What reciprocal impacts do lung bacteria and eukaryotic cells have in the lungs and other tissues? Whatever the answers to these questions, the gut and lung ecosystems (consisting of micro-organisms and host cells) clearly link nutrition, respiratory and digestive health and immune defenses, through an intricate system of reciprocal communication.

## Author Contributions

EM, UE-V, AR, and MT conceived the work and made major contributions to the writing of the manuscript. EM and UE-V made major contributions to the illustrations. DD, CC, PL, and SR contributed to the writing of the manuscript.

## Conflict of Interest Statement

The authors declare that the research was conducted in the absence of any commercial or financial relationships that could be construed as a potential conflict of interest.

## Abbreviations

AMPs: antimicrobial peptides
AMs: alveolar macrophages
BAL: bronchoalveolar lavage
CCL11: C-C motif chemokine 11
CFU: colony-forming unit
CLR: C-type lectin receptor
CO_2_: carbon dioxide
DC: dendritic cell
DNA: deoxyribonucleic acid
FOXP3: forkhead box P3
GF: germ-free
HDM: house dust mite
HFD: high-fat diet
IFNγ: interferon gamma
Ig: immunoglobulin
IL: interleukin
iNKT: invariant natural killer T cells
LPS: lipopolysaccharide
mRNA: messenger ribonucleic acid
NLR: NOD-like receptor
PAR: protease-activated receptor
PD-1: programmed cell death protein 1
pDCs: plasmacytoid dendritic cells
PD-L1: programmed death-ligand 1
PRRs: pattern recognition receptors
rRNA: ribosomal ribonucleic acid
SCFAs: short-chain fatty acids
SFB: segmented filamentous bacteria
SPF: specific pathogen-free
Th: T-helper
TLR: Toll-like receptor
T_reg_: regulatory T cell
